# The impact of AI feedback on the accuracy of diagnosis, decision switching and trust in radiography

**DOI:** 10.1371/journal.pone.0322051

**Published:** 2025-05-09

**Authors:** Clare Rainey, Raymond Bond, Jonathan McConnell, Avneet Gill, Ciara Hughes, Devinder Kumar, Sonyia McFadden

**Affiliations:** 1 Ulster University, School of Health Sciences, York St, Northern Ireland; 2 Ulster University, School of Computing, York St, Northern Ireland; 3 University of Salford, School of Health and Society, Manchester, United Kingdom; 4 Head – MLOps, Layer6 AI/School of Medicine, Stanford University, Toronto, Canada; Tuiuti University of Parana: Universidade Tuiuti do Parana, BRAZIL

## Abstract

Artificial intelligence decision support systems have been proposed to assist a struggling National Health Service (NHS) workforce in the United Kingdom. Its implementation in UK healthcare systems has been identified as a priority for deployment. Few studies have investigated the impact of the feedback from such systems on the end user. This study investigated the impact of two forms of AI feedback (saliency/heatmaps and AI diagnosis with percentage confidence) on student and qualified diagnostic radiographers’ accuracy when determining binary diagnosis on skeletal radiographs. The AI feedback proved beneficial to accuracy in all cases except when the AI was incorrect and for pathological cases in the student group. The self-reported trust of all participants decreased from the beginning to the end of the study. The findings of this study should guide developers in the provision of the most advantageous forms of AI feedback and direct educators in tailoring education to highlight weaknesses in human interaction with AI-based clinical decision support systems.

## Introduction

The current backlog and delay in the reporting of radiographs has driven investigations into the adoption of new technologies that could increase efficiency and “free up clinicians” to spend more time with patients [1,2]. Artificial intelligence (AI) has been proposed as a solution in automating the diagnosis of pathology on radiographic images, e.g., breast and chest imaging [3–5]. The dramatic developments in computer technology and processing ability have permitted ever more sophisticated and useful applications of AI. The latest technologies mimic the way the human brain functions, so that the AI can ‘learn’ from experience. AI systems have been shown to have a high degree of accuracy in the detection of abnormality on radiographic images, however clinical utilisation is incomplete due to the lack of transparency in how the system makes decisions resulting in trust issues between users and the system.

## Background

The first paper detailing the use of computers to assist in the diagnosis of pathology from radiographic images was published in the 1960s [6]. The rapidly increasing computational power available has permitted the development of ever more sophisticated pathology detection systems, such as the development and use of computer aided detection systems in mammography in the 1980s to proposals of autonomous triage systems in the present day [7]. Differing methods of analysis of radiographic, and other, images have been proposed. Deep Learning systems (DL) using Convolutional Neural Networks (CNNs) are one of the most recent and seemingly most promising forms of AI for detecting disease on radiographic images.

The use of AI has been targeted as an area of focus for modernising and future-proofing the NHS in the UK with proposed tasks such as image interpretation, autonomous triage and natural language processing [8]. This is particularly important in worldwide healthcare systems coping with the current and ongoing pressures of the COVID-19 global pandemic, where resources are limited [9].

Promising accuracies of DL using CNNs for detection of pathology from plain radiographs have been reported for chest imaging [3,4] and mammography [10], however, possibilities for determining diagnosis from skeletal radiographs have been less extensively investigated [11]. This is despite plain radiography being the initial modality of choice when imaging this area, with recent figures quoting that plain radiography has made up in excess of 23 million of a total of 44.7 million radiographic imaging examinations a year in the UK (in the period May 2018 to May 2019 alone) [12]. In the USA, the numbers of imaging examinations involving radiation continues to rise [13], although more detailed national data is not available.

The first publication of promising experimental results for detecting fractures on skeletal extremity radiographs was in 2017 [14]. Since then, other findings have been published evidencing the impressive performance of CNNs for pathology detection in comparison to, and in conjunction with, human experts [11,15–17].

Despite reported accuracies and benefits, clinicians’ trust in AI remains a barrier to AI implementation in the health care setting [7,18]. This is particularly the case with the use of DL systems. DL algorithms make use of multiple neural layers to analyse and process image data but there are a number of these layers which are hidden to the user. It is not entirely apparent, therefore, how the algorithm reaches its ultimate decision. This raises ethical and legal issues as well as having implications for the users’ trust in the system – if the user doesn’t fully understand how the AI has reached its decision, can the clinician be expected to assume ultimate responsibly for the outcome [19]? Additional information provided by the AI system, such as percentage confidence in diagnosis, triage recommendation and suggestion for further imaging have been proposed as other useful AI outputs [20].

Attempts are currently being made to make the DL decision-making process more transparent using visual representations to highlight the areas on the image that the AI is attending to, for example, attention/saliency heatmaps and regions of interest superimposed onto the radiograph [21,22]. It is proposed that a user may be able to calibrate their trust in the AI if the user can see the area/s on the image that the AI focussed on when making its decision.

Decision switching occurs when a decision-maker changes their initial image interpretation or diagnosis based on new information, or by assessing the same information from a different perspective. In the field of medical imaging, an overreliance on computer input has been found to cause errors of commission and omission [23,24]. This is known as automation bias and is defined as a human naively over-relying on computer information. This happens when the human has more faith in the machine rather than their own cognitive conclusions [23,24]. This would not be a problem in a perfect system, but this is not reflective of real life, where errors can occur in both humans and computer systems. Using AI may also cause the user to choose to change their mind in a positive direction, resulting in the desirable outcome of an increase in diagnostic accuracy as a result of interaction with the AI.

This study investigates the effect of feedback from an AI algorithm on diagnostic accuracy, decision switching and trust in student and qualified radiographers. The latest census from both the Royal College of Radiologists [25] and the Society and College of Radiographers [26] identify shortages of imaging professionals of up to 17% across the UK. With increased numbers of newly qualified and student imaging professionals in the NHS to fill this gap, it is important to understand how they, as well as currently practicing radiographers, will interact with new technologies being integrated into the imaging department. This study focuses on diagnostic radiographers, but these findings may be useful in benchmarking the impact of different forms of AI feedback on accuracy, decision switching and trust in all clinicians who use radiographic images for diagnosis.

To the authors’ knowledge, no study has investigated the impact of the type of AI feedback on the diagnostic accuracy of radiographers and the impact that level of experience has on the acceptability of the AI decision.

### Summary

AI is present and will be increasingly more present and utilised in healthcare moving into the future. This study aims to clarify how radiographers and student radiographers are affected by feedback from a poorly functioning AI system. This is particularly important as the literature is brimming with potentially promising results of AI performance. This study uses an AI which performed well in the laboratory (test set) but poorly with more clinically relevant images (clinical dataset). In addition, any difference in the perceived trust and acceptance of AI aided diagnoses between students and qualified radiographers was also investigated**.** Findings are intended to provide direction for educating undergraduate and practicing clinicians to maximise the promise and recognise the pitfalls of integrating AI into the clinical setting. It is envisaged these findings will provide an indication of the areas where caution should be exercised to aid developers to incorporate the most useful forms of AI feedback in their systems.

### Aim and objectives

This experimental study aimed to discover how a binary diagnosis and visual feedback from an AI algorithm affects the diagnostic accuracy of radiographers with differing levels of expertise when interpreting radiographic images of the upper extremities.

**Fig 1:**
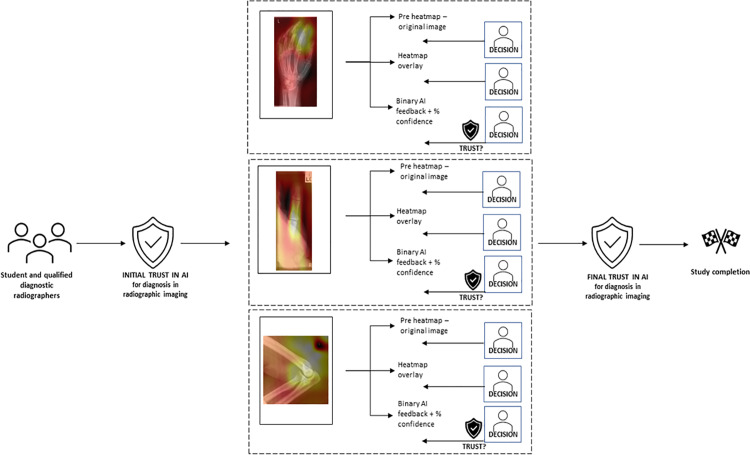
Graphical representation of study pathway.

The principal aim was to quantify the impact of performance, decision switching and trust in an AI algorithm following exposure to two different forms of AI feedback. Two forms of AI feedback were assessed for their respective impacts:

- AI feedback type 1) an attention map that shows where on the image the AI is attending to when making its decision (Fig 1) and
- AI feedback type 2): a simple binary diagnosis, i.e., the model suggests that there is either a pathology or no pathology (with a percentage confidence in its decision).

This study has the following objectives:

(i) to determine the baseline diagnostic accuracy of radiographers of differing levels of expertise when interpreting a selection of radiographic images of the upper appendicular skeleton.
(ii) to expose the participants to both binary and visual feedback from an AI algorithm.
(iii) to investigate the impact of the AI feedback on diagnostic accuracy.
(iv) to investigate the effect of this AI feedback on decision switching.
(v) to investigate the perceptions of trust of participants on the AI system.

## Methods

### Ethical approvals

Ethical permission for this study has been granted by Ulster University Nursing and Health Research Ethics Filter Committee FCNUR-20–035. Online, informed consent was gathered form all participants prior to commencement of the study, by an initial slide presentation, detailing the background, aims and objectives of the study. There were no minors participating in this study. Participants were permitted to exit the study at any point, but they were informed that their submissions up to this point would be included in analysis. Ethical permission for the use of the clinical dataset images has been granted previously to use the images for research purposes (Monash University, Clayton, Australia, 2011).

### Model training

MURA, a large dataset for abnormality detection in musculoskeletal radiographs, was used for training and testing of our AI model. MURA consists of 40,561 images taken by conducting 14,863 studies of the upper extremity. Each study is then labelled by a radiologist as either abnormal or normal. For this binary classification task, musculoskeletal radiographs from seven upper extremities including shoulder, humerus, elbow, forearm, wrist, hand and finger were used. The dataset is divided into training and validation sets with 9045 normal and 5818 abnormal radiographic studies divided between the two sets. In the training set, there are 11,184 patients with 13,457 studies and 36808 images. The images in all the sets vary in resolution and aspect ratio with no overlap of patients between training and validation set.

### Test set

As there was no explicit test set, we use half of the validation set (783 patients, 1,199 studies and 3197 images) as our test set and the rest as validation set. We made sure again that there was no overlap between any of the sets. The test set was chosen to contain approximately half of each of the upper extremities for adequate and balanced representation of each class.

### AI model

In this study we used a convolution neural network (CNN) specifically ResNet-152 pretrained on image net. During training time, one or more views of study is presented to the CNN and arithmetic mean of the output is taken to determine whether it’s abnormal or normal, similar to the original MURA study [40]. Any probability greater than 0.5 is deemed as abnormal. Using this criteria, the model is trained using the training set till the network stopped improving and training was shut off using early stopping criteria. For optimization, Adam optimizer was used with initial learning rate to 10^-4.

### Salience map

To understand the model output prediction and for use in this study, we create a binary saliency map for each image output alongside its abnormality score. Each salience map is created using the binary map creation technique as described by Kumar et al., 2018 [21]. In the binary saliency map, we use a heat map overlay in which the white indicates the strongest regions and black indicates null values. Spatial location of the binary saliency map (and the associated heat map) indicates the spatial area in the input radiography image which is used by the model to produce the given output. The strength of heat map indicates the strength of spatial regions that contribute the most towards the given output abnormality score. This form of explainable AI will allow the participant to determine if the binary output of the AI is related to the appropriate area of the image, or based on another area of the image that the users deem incorrect or inconsequential.

### Test dataset

The test dataset consists of radiographic images of the upper appendicular skeleton. They were obtained on real patients presenting to a hospital in Australia and were used as part of another PhD study [41]. The radiographic examinations in the dataset have all been anonymised. All patients’ identifiable information such as the patient’s name, date of birth and health and care number have been removed from each image. Images do not contain any rare abnormalities or pathologies which could readily identify an individual.

There are a total of 268 examinations in the full dataset and have approximately a 3:7 split of pathology: no pathology. 21 examinations were chosen at random for inclusion in this study.

The participants will be blinded to the ground truth at all stages of the study, to avoid bias [42].

The radiographic examinations have been used to determine diagnosis. There are three to five radiological reports from radiologists and reporting radiographers available for each. Consensus binary diagnosis has been determined by inspection of radiology reports (fracture/no fracture), and this consensus is used as ‘ground truth’ in this study. Agreement of the participants in this study with ‘ground truth’ has been termed ‘accuracy’.

The AI model described above was used to obtain diagnosis for each examination. Predictions were produced as a binary diagnosis (i.e., pathology/no pathology) and percentage confidence of the AI in its decision. A heatmap overlay (GradCAM) was also provided on each image (Fig 6).

Of the 21 examinations included in this study, the AI made the correct prediction on 12 examinations (57.1% accuracy). There was pathology present on nine out of the 21 examinations (42.9%) (Available in ‘Supporting Information’, S1)

### Patient-public involvement

A PPI group was set up to help drive the direction of this study and to ensure the study is relevant and useful to the public and radiographers in clinical practice. The group consisted of two student radiographers, two practicing radiographers of differing levels of experience (approximately 40 years’ and 15 years’ experience) and one patient with a clinical history of repeated attendance for plain radiographic examinations of the appendicular skeleton due to repeated sports injuries.

### Pilot study

Six images were selected randomly from each anatomical region in the test dataset (fingers, hand, wrist, forearm, elbow and humerus) and embedded into Qualtrics® for interpretation by seven participants. Purposive sampling was used to select participants to the pilot study who represent the target respondents, to ensure all potential participants would understand expectation of their input. Representation from each year of a UK diagnostic radiography programme (Ulster University) was obtained, along with qualified radiographers with differing lengths of clinical experience. Participants were asked to comment on the acceptability of the study design, the quality of the images in the survey and the time taken to complete the survey, ensuring face and content validity and the acceptance of the time sacrifice required to complete the study. This information was used to build the survey for the full study.

### Qualtrics survey

The number of images in the survey was chosen based on an acceptable estimated time for completion (approximately 15 minutes). From the test dataset (n=21 examinations) the randomiser function in Qualtrics was used to allocate three radiographic examinations to each participant. Three examinations were chosen to encourage participation as the time taken to complete is deemed to be acceptable to participants and encourage thoughtful responses, therefore avoiding random responses and premature cessation of the survey [43].

Each examination contained two or more radiographic images. Each image in the examination was presented, and the participant was asked to determine if there was a pathology present on the image. The participant was then presented with the heatmap overlay and asked again if they felt there was a pathology present, and whether the AI heatmap has caused them to change their mind from their initial decision. This was repeated for each image in the examination. When all images had been presented, the participant was presented with the images again and asked if they felt there was a pathology present. The responses to this question were not included in the analysis of the data but was provided to ensure the participant had access to all images again to best determine the impact of the binary feedback, which was determined for the entire examination and not per image.

Following the above, participants were given the AI binary diagnosis and asked if they would like to change their mind from the first evaluation of the images. When all images, heatmaps and binary diagnoses were presented, the participants were asked to determine if they felt there was a pathology present. This question was included to represent the clinical scenario, where clinicians would have the opportunity to view all images to determine a final diagnosis. They were then provided with the AI binary decision and asked if they now believed there to be a pathology present on the image. They were asked if the binary feedback caused them to change their mind from their initial decision and to give indication in their trust in the AI following exposure to all images and AI feedback for this examination (Available in ‘Supporting Information’).

### Participant selection

The study was open to all diagnostic radiographers, who are currently in clinical practice, including students. The landing page of the Qualtrics® survey provided participants with information on the study rationale and aim. A brief precis of the relevant literature on the subject was also provided. Informed consent was requested by indication of the participants desire to proceed via a yes/no response. If the participant indicated that they did not give their consent the ‘skip logic’ function exited them from the study. A final page notified respondents of submission of responses, although a full review of responses was not given. The study was promoted via the European Congress of Radiology (ECR) Research Hub (open from 2^nd^ March to the 12^th^ April 2021) and by promotion on social media (Twitter® and LinkedIn®). The last response included in analysis was collected on the 2^nd^ November 2021. Data was, therefore, collected between 2^nd^ March to 2^nd^ November 2021. Due to the lack of research in the area, this method of convenience, snowball sampling was felt to be appropriate to gain insight upon which to base future studies. A power calculation was not carried out due to the lack of previous studies in this area, however, ‘rule-of-thumb’ estimates indicate that there should be 10–15 participants in each group for quantitative studies [44,45].

Participants were grouped according to broader experience groups in order to ensure adequate sample size in each group (student radiographers/qualified radiographers) to allow for more meaningful outcomes from statistical analyses.

### Statistics and reproducibility

Tests of normality (Kolmogorov-Smirnov and Shapiro-Wilk) were conducted. Skewness and kurtosis were visually determined by inspection of histograms and distribution curves. Comparison was made of the mean and median for each condition in both the student and radiographer groups. Data was found to be normally distributed and parametric tests were used for inferential statistics (Table 2).

**Table 1:** Demographic details of participants.

Demographic information		
**Currently practicing plain radiography in your role (student or radiographer)?**	*Yes*	100% (n=94)
	*No*	0% (n=0)
**Gender**	*Male*	30.9% (n=29)
	*Female*	69.1% (n=65)
**Age range**	*18-25 years old*	53.2% (n=50)
	*26-35 years old*	23.4% (n=22)
	*36-45 years old*	16% (n=15)
	*46-55 years old*	5.3% (n=5)
	*55-65 years old*	2.1% (n=2)
**In which country do you currently work/study?**	*England*	8.5% (n=8)
	*Ireland*	3.2% (n=3)
	*Italy*	1.1% (n=1)
	*Jordan (JO)*	1.1% (n=1)
	*Malta*	5.3% (n=5)
	*Northern Ireland (NI)*	52.1% (n=49)
	*Philippines*	1.1% (n=1)
	*Portugal*	3.2% (n=3)
	*Scotland*	2.1% (n=2)
	*Sri Lanka*	1.1% (n=1)
	*United Arab Emirates (UAE)*	1.1% (n=1)
	*United Kingdom (UK)*	19.1% (n=18)
	*United States of America (USA)*	1.1% (n=1)
**Please select from the options below to indicate your level of experience in Diagnostic Radiography**	*Undergraduate student - year 1*	24.5%(n=23)
	*Undergraduate student - year 2*	13.8%(n=13)
	*Undergraduate student - year 3*	18.1%(n=17)
	*Undergraduate student - year 4 (Scotland only)*	1.1%(n=1)
	*TOTAL students*	57.5% (n=54)
	******************************************************	******************
	*	*
	*Less than or equal to 1 year experience*	3.2%(n=3)
	*Greater than or equal to 1 to less than 6 years’ experience*	4.3%(n=4)
	*Greater than or equal to 6 to less than 11 years’ experience*	13.8%(n=13)
	*Greater than or equal to 11 to less than 20 years’ experience*	11.7%(n=11)
	*Greater than or equal to 20 years’ experience*	9.6% (n=9)
	*TOTAL radiographers*	42.6% (n=40)
**How proficient would you consider yourself to be in the use of information technology (I.T.) in general**	*Very proficient: I choose to use IT and computer systems in all aspects of my personal and work life and feel comfortable with the introduction of newer systems.*	37.2% (n=35)
	*Proficient: I choose to use IT and computer systems in many aspects of my personal and work life, and I am somewhat comfortable with the introduction of newer systems.*	53.2% (n=50)
	*Somewhat proficient: I use IT and computer systems when I need to in my personal and work life, but I feel overwhelmed and confused by newer systems.*	9.6% (n=9)
**How are you accessing this survey?**	*Home personal computer (PC)*	42.6% (n=40)
	*Diagnostic display workstation*	3.2% (n=3)
	*Mobile phone*	42.6% (n=40)
	*Tablet*	5.3% (n=5)
	*Other*	6.4% (n=6)

**Table 2:** Test of normality.

	ALL (i.e., stud and rad)	Students	Radiographers
**Kolmogorov-Smirnov**	0.200 (significant, therefore indicating normal)	0.200	0.200
**Shapiro-Wilk**	0.489 (significant, therefore indicating normal)	0.158	0.564
**Sk**ewness	0.517 (std error 0.501)	0.917 (std error 0.501)	-0.920 (std error 0.501)
**Kurtosis**	0.342 (std error 0.972)	0.550 (std error 0.972)	-0.181 (std error 0.972)

Descriptive statistics are used to describe the impact of the AI feedback on participants’ accuracy. This is further sub-divided into experience categories (i.e., student and radiographer) and condition (i.e., instance where the AI was correct, incorrect, pathological cases and non-pathological cases). Data is presented per examination as each examination had differing numbers of images contained within. Participants were allocated three examinations at random, therefore data is analysed as % accuracy, rather than total number of decision points, however this data pertaining to the total number of decision points is given in Table 3.

**Table 3:** Impact of A.I feedback on student and qualified diagnostic radiographers’ diagnostic accuracy.

	Condition	AI feedback	Total participant decisions^*^	Total correct participant decisions^*^	% Accuracy	Standard deviation
**ALL**	Overall impact of AI feedback	No AI feedback	746	393	52.2	19.24
		AI heatmap	742	383	52.8	19.33
		AI binary feedback	245	149	60.6	25.06
	AI correct	No AI feedback	491	245	49.2	17.38
		AI heatmap	489	245	49.2	18.57
		AI binary feedback	153	97	62.6	21.69
	AI incorrect	No AI feedback	225	148	62.3	20.45
		AI heatmap	253	138	58.8	20.28
		AI binary feedback	92	52	57.5	31.13
	Pathological cases	No AI feedback	342	198	62.7	21.66
		AI heatmap	339	186	58.5	21.97
		AI binary feedback	104	73	70.3	27.73
	Non-pathological cases	No AI feedback	404	195	47.8	15.1
		AI heatmap	403	197	48.6	16.8
		AI binary feedback	141	76	53.4	21.61
**Student radiographers**	Overall impact of AI feedback	No AI feedback	417	207	49.9	23.08
		AI heatmap	432	193	45.6	22.92
		AI binary feedback	143	78	54.3	31.29
	AI correct	No AI feedback	271	126	44.3	22.79
		AI heatmap	287	119	40.8	21.51
		AI binary feedback	90	52	57.3	28.24
	AI incorrect	No AI feedback	146	81	59.0	21.9
		AI heatmap	145	74	53.3	24.44
		AI binary feedback	53	26	49.4	37.24
	Pathological cases	No AI feedback	192	116	63.3	22.77
		AI heatmap	208	98	50.7	30.66
		AI binary feedback	64	38	59.9	37.97
	Non-pathological cases	No AI feedback	225	91	39.9	18.31
		AI heatmap	224	95	41.7	15.32
		AI binary feedback	79	40	50.0	26.2
**Qualified radiographers**	Overall impact of AI feedback	No AI feedback	312	170	57.4	23.07
		AI heatmap	310	169	57.5	25.85
		AI binary feedback	102	66	64.9	34.33
	AI correct	No AI feedback	203	104	50.5	16.21
		AI heatmap	202	109	52.6	25.23
		AI binary feedback	63	44	65.2	28.32
	AI incorrect	No AI feedback	109	66	68.6	28.98
		AI heatmap	108	60	65.5	26.46
		AI binary feedback	39	22	64.3	44.63
	Pathological cases	No AI feedback	133	82	68.4	28.05
		AI heatmap	131	74	64.5	30.07
		AI binary feedback	40	34	84.8	22.8
	Non-pathological cases	No AI feedback	179	88	49.2	14.89
		AI heatmap	169	95	52.2	22.05
		AI binary feedback	62	32	49.9	34.58

* N.B. % agreement is calculated based on the % accuracy of each decision and therefore there is a slight discrepancy between this and the calculation based on columns two and three above.

**Fig 2:**
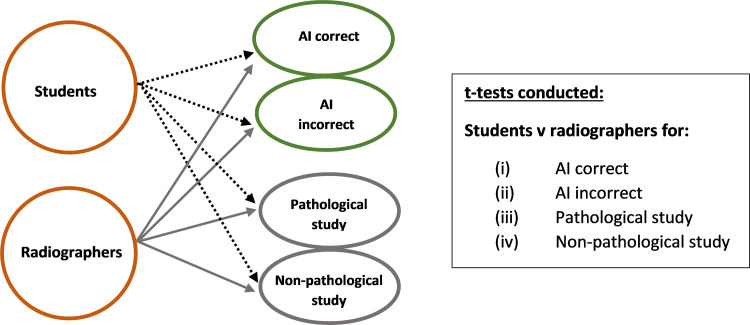
Graphical representation of data analysis – t-test.

Participant accuracy was not considered as related to the individual, but rather as a group: student or radiographer (Fig 2). Diagnostic accuracy was determined at three points; before any AI feedback, following exposure to the AI generated heatmap and following the AI binary diagnosis. The findings are tabulated, in full, in Table 3.

**Fig 3:**
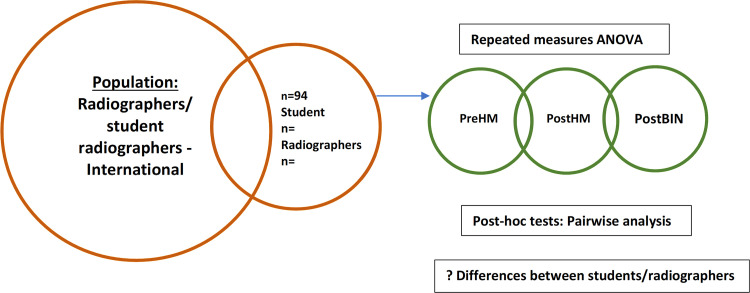
Graphical representation of data analysis – ANOVA.

The feedback was provided in a sequential manner, i.e., pre-heatmap (no AI feedback), post-heatmap and post-AI binary diagnosis, therefore repeated measured ANOVA was used to investigate the impact of the type of AI feedback provided (Fig 3). Post-hoc pairwise comparisons were conducted to determine the specific factors responsible for the differences. Combined effects of experience level (students, radiographers) were used investigate any differences in accuracy in response to the AI feedback. Effect size of any statistically significant finding was estimated using partial eta squared (*= SSeffect/ (SSeffect + SSerror)*). Effect sizes are reported using an established ‘rule of thumb’:

ηp2 = 0.01 indicates a small effect

ηp2 = 0.06 indicates a medium effect

ηp2 = 0.14 indicates a large effect [46]

T-tests (two-tailed) investigated the significance of any differences between the accuracy of the student and radiographer groups under each of the four investigated conditions: (i) AI correct, (ii) AI incorrect, (iii) pathological cases and (iv) non-pathological cases. Cohen’s *d* was used to estimate effect size of any statistically significant result: small 0.2, moderate 0.5, large 0.8 effect [47].

Repeated measures ANOVA was used to investigate any statistically significant difference between the impact of the type of AI feedback and diagnostic accuracy. The rate of decision switching has been presented using descriptive statistics for the collective group for each of these scenarios: where the (i) AI was correct, (ii) AI was incorrect, and where the image was (iii) pathological or (iv) non-pathological. This was repeated for each group (students and radiographers). The direction of the switch of each of the groups in each of the conditions (as before: (i)-(iv)) through the impact of the AI on accuracy, where if the accuracy of the group increased, the AI feedback had a positive effect on the diagnostic accuracy of the participants.

Data was tabulated and graphically represented, where appropriate.

The impact of the different forms of AI feedback on the propensity of the participants to change their mind from their initial diagnosis were investigated. All participants were asked if the AI feedback caused them to change their mind from their initial diagnosis. This question was posed following the AI feedback in the form of the heatmap and again following provision of the AI binary diagnosis.

## Results

All data analysis was conducted on SPSS® v 27 [27] and Microsoft® Excel® [28].

### Demographics

Full demographic details of the participants are given in Table 1. Following cleaning of the data there were 94 participants included in the analyses. Responses were included if at least part of the study was completed. Responses were removed if the participant did not give consent via the Qualtrics platform or did not complete any part of any of the questions. Of the 94 participants, 57.5% (n=54) were students and 42.6% (n=40) were radiographers with representation of a range of experience levels from year one of an undergraduate degree programme to greater than 20 years clinical experience. Most respondents were from the UK (England, Scotland, Northern Ireland) or Ireland (85%, n=80).

### Accuracy

**Fig 4:**
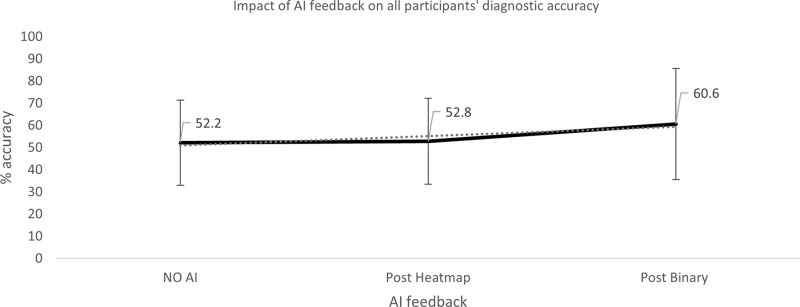
Impact of AI feedback on *all* participants’ diagnostic accuracy.

**Fig 5:**
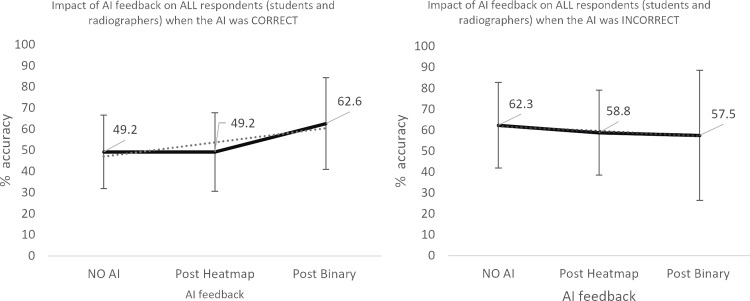
Impact of AI feedback on *all* participants’ diagnostic accuracy, when the AI feedback is *correct* and *INcorrect.*

**Fig 6:**
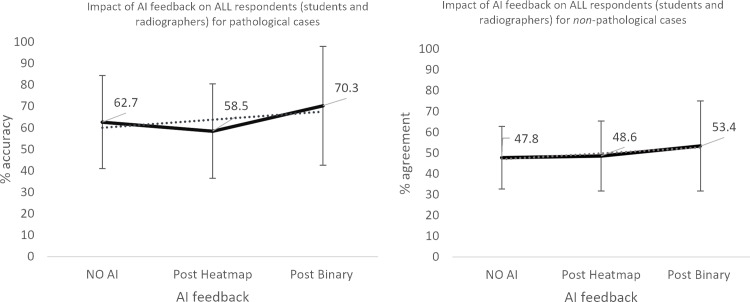
Impact of AI feedback on *all* participants’ diagnostic accuracy in *pathological* and *non-pathological* cases.

**Fig 7:**
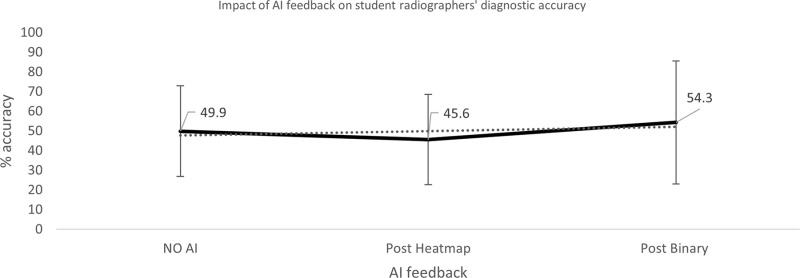
Impact of AI feedback on *student* participants’ diagnostic accuracy.

**Fig 8:**
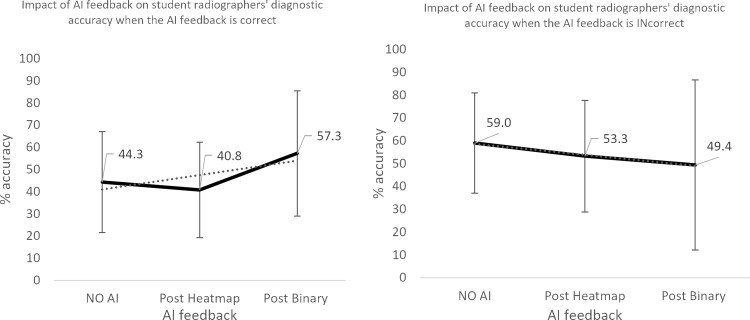
Impact of AI feedback on *student* participants’ diagnostic accuracy, when the AI feedback is *correct* and *INcorrect.*

**Fig 9:**
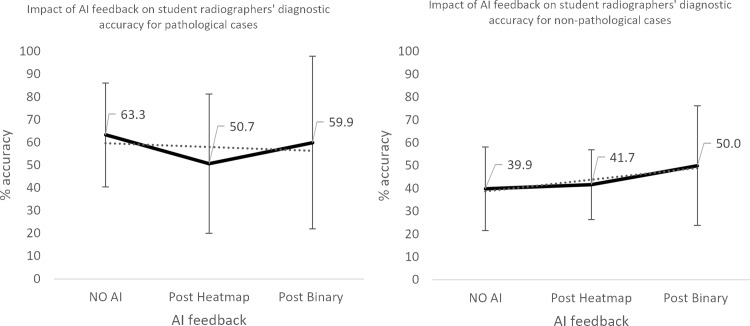
Impact of AI feedback on *student* participants’ diagnostic accuracy in *pathological* and *non-pathological* cases.

**Fig 10:**
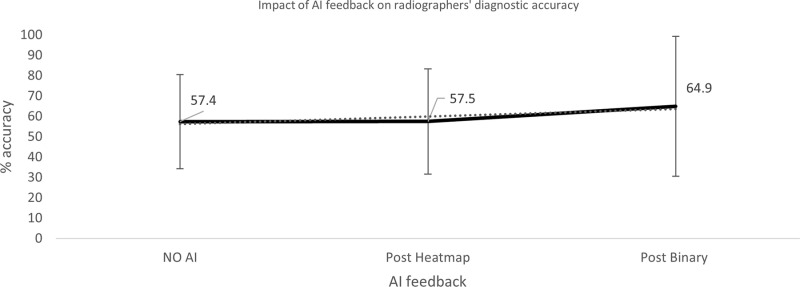
Impact of AI feedback on *radiographer* participants’ diagnostic accuracy.

**Fig 11:**
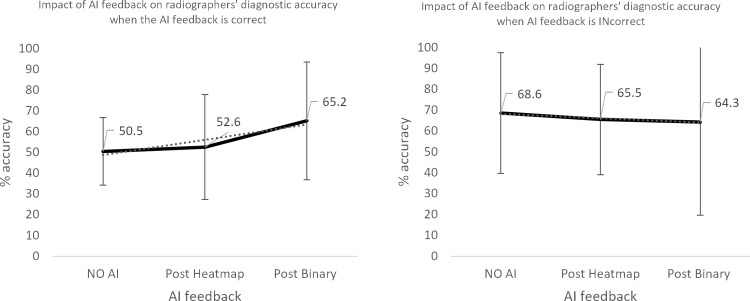
Impact of AI feedback on *radiographer* participants’ diagnostic accuracy, when the AI feedback is *correct* and *INcorrect.*

**Fig 12:**
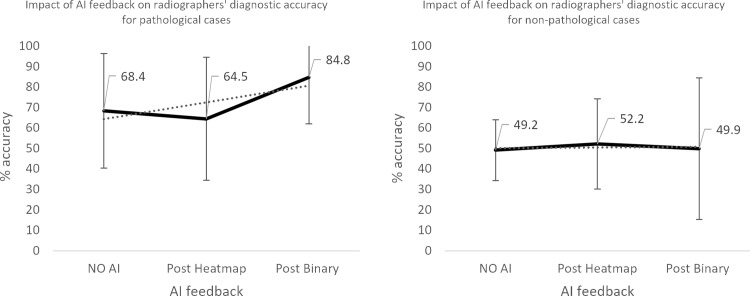
Impact of AI feedback on *radiographer* participants’ diagnostic accuracy in *pathological* and *non-pathological* cases.

‘Ground Truth’ has been determined as consensus diagnosis from at least three out of five reporting radiographers and radiologists (see Methodology section). ‘Accuracy’ is defined in this study as the agreement of the participants with the ground truth diagnosis. Percentage accuracy in each of the two experience levels – student radiographer (‘students’) and qualified radiographers (‘radiographers’) is reported. Descriptive statistics are reported for each experience level. Data was found to be normally distributed (see Methodology section, and Table 2), and further analysis of the significance of any relationships in the data are reported using t-tests (α=0.05), comparing accuracy of student and radiographer groups and repeated measures ANOVA, investigating the impact of each form of AI feedback (Fig 2 and 3). Full results are presented in Figs 4–12 and Tables 3–5. Dotted lines in all figures represent lines of best fit/ trendline.

The qualified radiographers had a greater accuracy across all examinations, under all conditions, however, this difference was not statistically significant. Initial accuracy was used as a baseline to clarify the impact of the AI feedback (Figs 4–12). The standard deviation is presented as error bars on all graphs and listed numerically in Table 3. These are large as there are differing accuracies across all examinations, i.e., some examinations may be more ‘difficult’ to interpret than others, although ‘difficulty’ of the task was not included in the analysis for this study. Figs 4–12 illustrate the impact of the AI feedback on participants collectively, followed by more granular analysis of students and radiographers under each of the four conditions – when the AI agrees with ground truth (‘AI correct’), disagrees with ground truth (‘AI incorrect’), in pathological cases and non-pathological cases. The initial point on the graph represents the initial accuracy of the users collectively and further points illustrate the impact of the heatmap form of feedback and binary, sequentially.

Further interrogation of the data revealed that although there was no statistically significant difference in the two groups’ diagnostic accuracy, there was a small to moderate effect size under all conditions – when the AI feedback was correct, when the AI was incorrect, in pathological cases and in non-pathological cases. This disparity between statistical significance and effect size may be due to small sample size in some cases (n=16–26). The findings are presented in full in Table 4.

**Table 4:** t-tests comparing students and radiographers’ accuracy in determining diagnosis from radiographic images, following AI decision support, across four conditions: AI correct/incorrect and pathological/non-pathological cases.

Comparison *(student (stud) v radiographer (rad) for…)*		Mean *(M=…)*	Std Dev *(SD=…)*	Levene’s test *(greater than 0.05 – equal variance between groups assumed)*	t	Sig *(p=…(two-tailed))*	Magnitude of difference in means *(mean diff=…)*	95% CI:…	Cohen’s d=… *(small 0.2, moderate 0.5, large 0.8 effect (Pallant, 2009)*
**…ALL examinations (n=42)**	Stud	47.2886	22.45583	0.974	-1.554	p=0.128	-10.95571	-25.20019 to 3.28876	-0.480 CI: -1.091 to 0.137
	Rad	58.2443	23.21394						
**…instances where the AI was correct (n=26)**	Stud	43.7354	21.77342	0.765	-1.184	p=0.248	-9.55692	-26.21770 to 7.10386	-0.464 CI: -1.239 to 0.320
	Rad	53.2923	19.31483						
**…instances where the AI was INcorrect (n=16)**	Stud	53.0625	23.80118	0.424	-1.019	p=0.325	-13.22875	-41.06032 to 14.60282	-0.510 CI: -1.499 to 0.497
	Rad	66.2912	27.939115						
**…pathological cases (n=18)**	Stud	54.0689	28.5624	0.659	-1.097	p=0.289	-14.69444	-43.09079 to 13.70190	-0.517 CI: -1.450 to 0.431
	Rad	68.7633	27.86386						
**…NON pathological cases (n=24)**	Stud	42.2033	15.53152	0.568	-1.265	p=0.219	-8.15167	-21.51086 to 5.20753	-0.517 CI: -1.325 to 0.303
	Rad	50.3550	16.02225						

### Impact of AI feedback

**Fig 13:**
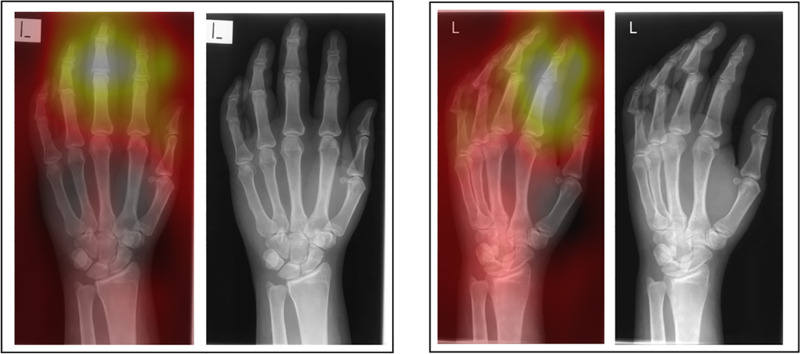
Patient 11 – Pathological examination: AI correct (83.6% confidence).

**Fig 14:**
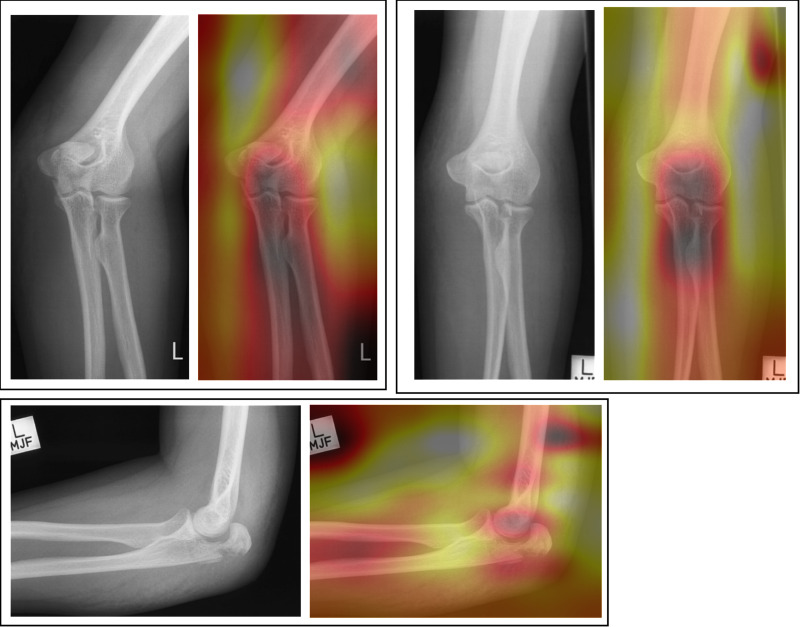
Patient 2 – Pathological examination: AI incorrect (99.3% confidence in *incorrect* diagnosis).

**Fig 15:**
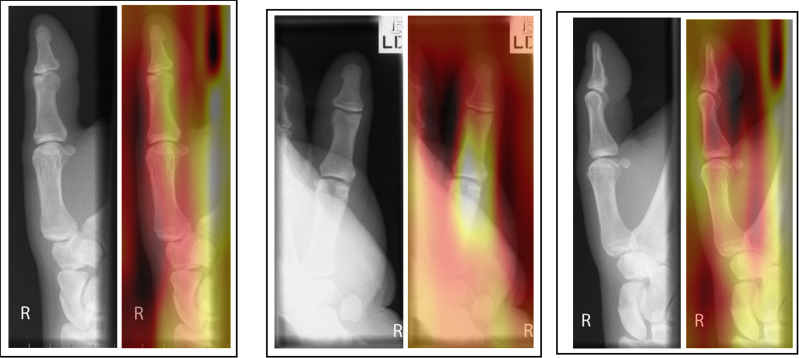
Patient 16 – Non-pathological examination: AI correct (97.0% confidence).

**Fig 16:**
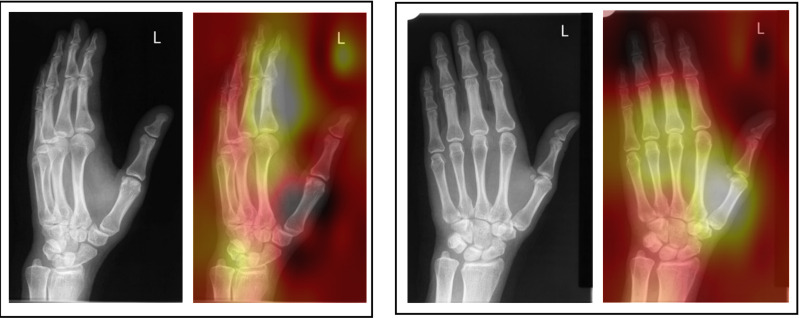
Patient 12 – Non-pathological examination: Ai incorrect (65.24% confidence in *incorrect* diagnosis).

Two forms of AI feedback were provided in sequence in this study – 1) a ‘heatmap’ overlay and 2) binary diagnosis with % confidence from the model in its diagnosis. The heatmap (or ‘saliency’ map) provides a visual indication of the area/s of the image that the system found most important in determining its diagnosis (Figs 13–16).

There is a statistically significant difference in participant accuracy following AI feedback (i.e., pre-AI feedback, post-heatmap and post-binary feedback from the AI) when the AI is correct (*p=*.002) and when the examination has been determined as demonstrating pathology (*p=*.013) (Available in ‘Supporting Information’).

Pairwise comparisons indicate that when the AI is correct there is a significant improvement in the participants’ performance before presentation with any AI feedback and following presentation of the binary AI feedback (*p=*.007) (i.e., between the ‘plain’ image and following textual AI feedback: ‘*The AI system determined that this examination/imaging series DID/DID NOT contain evidence of pathology with x % certainty’*). In the case of pathological examinations, there was a statistically significant difference between both the pre-AI feedback and post-heatmap stages (*p=*.015) and post heatmap and post binary feedback (*p=*.013). Further inspection of the descriptive statistics would indicate that there was a statistically significant decrease in performance following presentation of the heatmap, followed by an increase, exceeding the performance with the un-aided interpretation (No AI feedback 65.85%, post-heatmap 57.62%, post-binary feedback 72.35%), indicating that the heatmap was detrimental to performance in pathological cases.

### Decision switching

**Fig 17:**
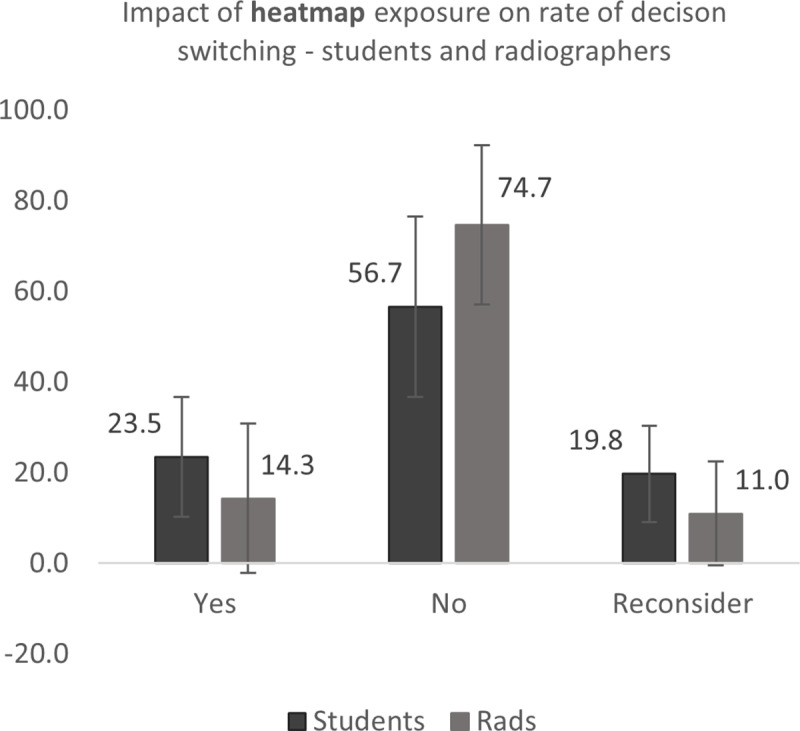
Impact of *heatmap* feedback on students and radiographers’ propensity to change their mind from their original decision.

**Fig 18:**
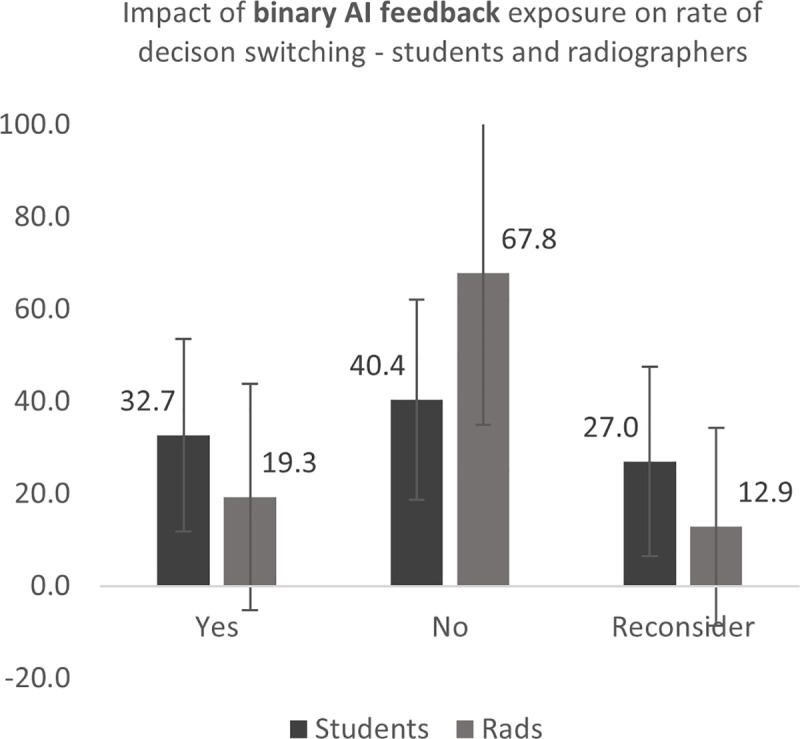
Impact of *binary* feedback on students and radiographers’ propensity to change their mind from their original decision.

Students were more likely than radiographers to change their mind following heatmap feedback (23.5% students, 14.3% radiographers – difference 9.2%) (Fig 17 and 18). The student group were also more likely to change their mind following binary feedback, with a greater difference between the two experience groups than heatmap provision only (32.7% students, 19.3% radiographers – difference 13.4%). There was also a difference found in the instances where participants felt they would reconsider their initial opinion following both heatmap and binary diagnosis (19.8% students, 11.0% radiographer – difference 8.8%; 27.0% students, 12.9% radiographers – difference 14.1%, for heatmap and binary AI feedback respectively) (Figs 17 and 18). This indicates that the AI feedback is more likely to cause students to change their mind from, and feel uncertainty in, their initial decision.

The Mann-Whitney *U* test was conducted to investigate any statistical significance of these findings. The decision switching rate of student radiographers differed significantly from radiographers following presentation of the heatmap, for yes (p=.023), no (p=.002) and reconsider responses (p=.008), with the student group responding that they changed their mind or reconsidered their initial diagnosis more often that the radiographer group. The radiographer group responded that they did not change their mind following their initial decision more often than students following both heatmap and binary AI feedback. A medium effect size (r = $\frac{\text{z}}{\surd n}$) was found in all cases. Full results are presented in Table 5a.

**Table 5a:** Mann Whitney U test applied to differences in rates of decision switching (instances of yes, no and reconsider expressed as a proportion of the total reponses) of students and radiographers. Mean ranks are reported and effect size has been repoting using Pearson’s r with effect sizes: small 0.1-0.3, medium 0.3-0.5 and large 0.5 and over (Cohen, 1988).

	*Has being given the AI feedback (heatmap or binary AI decision) caused you to change your mind from your initial diagnosis?*	Student n=21/ Radiographer n=20 *Total n = 41*	Mean rank	Mann-Whitney U	z=…	Exact significance (*p=…)*	Effect size: r = $\frac{\text{z}}{\surd n}$
Following heatmap	*Yes*	Student	25.10	124.00	-2.257	.023	0.35 (medium)
		Radiographer	16.70				
	*No*	Student	15.43	93.00	-3.055	.002	0.48 (medium)
			Radiographer	26.85			
	*No, but it did make me reconsider my initial decision*	Student	25.74	110.50	-2.604	.008	0.41 (medium)
			Radiographer	16.02			
Following binary AI diagnosis	*Yes*	Student	24.62	134.00	-2.017	.044	0.32 (medium)
		Radiographer	17.20				
	*No*	Student	15.67	98.00	-2.934	.003	0.46 (medium)
			Radiographer	26.60			
	*No, but it did make me reconsider my initial decision*	Student	25.55	114.50	-2.571	.009	0.40 (medium)
			Radiographer	16.23			

As this data was self-reported by the participants, further analysis was conducted on the respondents’ diagnosis to determine the rate and direction of the decision switch, i.e., whether their change of mind was positive (switching from an incorrect decision to a correct one) or negative (Table 5, Figs 17 and 18). The direction of the switch was noted as *positive*, i.e., more correct, and *negative*, i.e., less correct, and *no change*, where the group of participants did not change their minds. Data was, again, analysed collectively for the two groups (students and radiographers) as the number of decision points varied across the participants. The direction of the switch was determined for three comparisons: (i) pre and post heatmap (i.e., impact of heatmap only), (ii) pre heatmap and post binary feedback (the effect of all AI feedback) and (iii) post heat map and post binary (effect of binary feedback only).

### Automation bias

Automation bias was investigated by determining the negative impact of each type of feedback. The student group was more likely to change their mind to a more incorrect response, following AI feedback. Figs 4 to 12 represent the impact of each type of AI feedback on the accuracy of participant interpretation. Additional analysis of the direction switch is given in Table 5b, by subtracting the initial and final diagnostic accuracy of the participants. The AI feedback (i.e., heatmap and binary AI decision) proved beneficial to participants except for situations where the AI was incorrect and pathological examinations in the student group (decrease in accuracy of 3.4%). This effect was greater in the student group (9.6% decrease).

**Table 5b:** Decision switching before any AI feedback and after all AI feedback, reported as % difference in diagnostic accuracy of participants (i.e., difference in diagnostic accuracy before AI feedback and diagnostic accuracy after all AI feedback). Grey highlighted cells represent instances where the AI feedback had net negitive impact on diagnostic accuracy for the examination.

	Condition	Difference (Before AI – after all AI feedback)
**ALL**	AI correct	+13.4
	AI incorrect	-4.8
	Pathological examinations	+7.6
	Non-pathological examinations	+5.6
**Students**	All AI feedback	+4.4
	AI correct	+13.0
	AI incorrect	-9.6
	Pathological examinations	-3.4
	Non-pathological examinations	+10.1
**Radiographers**	All AI feedback	+7.5
	AI correct	+14.7
	AI incorrect	-4.3
	Pathological examinations	+16.4
	Non-pathological images	+0.7

### Trust analysis

Trust perception (0 representing no trust and 5 representing absolute trust) has been gathered at several points during the study:

- At the beginning of the study, when participants had no access to any of the images nor AI feedback provided as part of this study.
- Following exposure to all images, heatmap and binary feedback in each complete examination, i.e., three per participant
- Finally, at the end of the study, when the participant will have engaged with the full study, consisting of three complete examinations including all images and AI feedback contained therein

(Table 6, Fig 19).

**Table 6:** Trust perception of student radiographers and radiographers at the beginning of the study, before accessing any of the AI feedback, following exposure to the AI and at the end of the study, following exposure to all examinations and all AI feedback associated with the allocated examinations.

	Student			Radiographer		
	n=	Mean	SD	n=	Mean	SD
**Trust perception - START**	54	4.1	0.9	40	3.9	1.1
**Trust perception - DURING**	142 (total perception ratings – three examinations per participant)	3.5	0.6	101 (total perception ratings – three examinations per participant)	3.0	0.7
**Trust perception – END**	44	3.4	1.3	34	3.2	1.1

**Fig 19:**
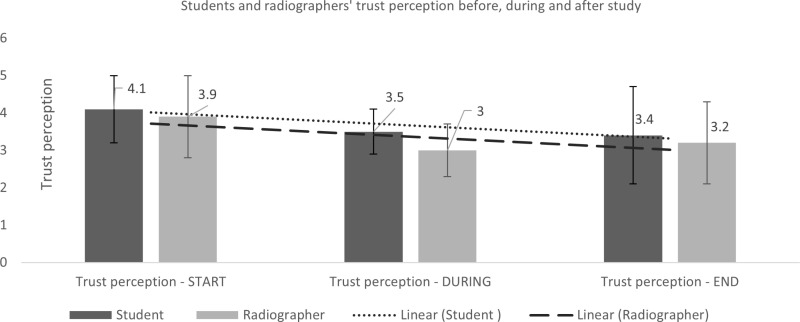
Students’ and radiographers’ trust perception before, during and after AI feedback.

This data is analysed using descriptive statistics, firstly to determine the differences in mean trust of all participants at the beginning, following each examination and again at the end of the study and secondly, sub analysis of the two groups: students and radiographers.

Initial mean trust is lower for the radiographer group than the student group (mean=4.1, n=54, SD 0.9; mean=3.9, n=40, SD 1.1, for students and radiographers respectively). Trust at the end of the study, compared to the beginning, decreased in both groups (mean=3.4, n=44, SD 1.3; mean=3.2, n=34, SD 1.1, a decrease of 0.7 for both students and radiographers respectively). Overall, mean trust is higher in the student group than the radiographer group during the image assessments, i.e., when asked after each heatmap and each AI binary feedback (3.5, n=142, SD=0.6; 3.0, n=101, SD=0.7 for students and radiographers respectively).

### Accuracy

The level of diagnostic accuracy was lower than expected overall and does not compare with performance reported in recent literature [29,30]. Participants were accessing the study on personal devices which would not permit the optimal viewing conditions available in the clinical setting. The images were presented ‘one at a time’, which although reflects how images are acquired in the clinical setting, does not permit the participant to revisit a previous image until the end of the examination. This is unlike the clinical scenario, where the radiographer would refer to all images when making a decision. This was intentional to attempt to glean insight into the impact of the different forms of feedback offered.

There was no statistically significant difference in the diagnostic accuracy between students and radiographers in this study, although radiographers were more accurate in their diagnosis across all conditions (AI correct/incorrect, pathology/no pathology). Each examination was different, and the difficulty of diagnosis may have had an impact of the relatively low level of accuracy in places. In this study radiographers and students were grouped together irrespective of the amount of clinical experience they had. This may in part explain these findings as newly qualified radiographers may have diagnostic accuracy like final year students. Supporting this, amongst the participants in this study there is greatest representation in the ‘greater than or equal to six, but less than 11 years’ experience group’ (Table 1). Other studies have investigated the impact of computer feedback on user performance but have further categorised experience level of the participants. Goddard et al. (2014) [23] investigated computer-assisted decision support in medicines prescribing and found that automated decision support improved the accuracy of all participants, independent of experience. The accuracy of the AI also did not seem to have an impact on either students or radiographers. However, on all occasions (inaccurate and accurate AI feedback), the heatmap caused a decrease in accuracy before presentation of the AI binary decision rectified the loss in performance, which often surpassing the initial accuracy. The accuracy of the AI (correct or incorrect) did not affect this increase in performance.

The necessity for the use of visual forms of AI explainability have been mooted by clinical professionals [31,32]. Opinion is changing from explainability being central to the successful adoption of AI, to some questioning its value [33]. A recent study [31] investigated the agreement of area of pathology and the area identified by a number of different types of AI heatmap. The study found some forms of heatmap (GradCAM) were broadly similar to the area identified by human experts but noted that all heatmaps tested used were ‘coarse’ and lacking in detail. They concluded that the heatmaps tested were not yet precise enough to be relied upon for diagnostic assistance or explainability. This may explain why the heatmap caused some degree of confusion in this study, even in instances where the binary diagnosis from the AI was correct. This study supports the recognition that any form of explainability should be treated carefully and the impact of using differing forms of AI explainability should be carefully researched before clinical adoption. This study only investigated the heatmap form of visual explainability and therefore further study should investigate if different visual representations of the focus of the AI might be of better use in this technologically proficient profession. Previous work by Rainey et al., 2021b, 2022a and 2022 [20,34,35] have reported that the preference of this population (radiographers) may be for the AI to provide data relating to the accuracy of the system being used and a degree of confidence of the system in making its diagnosis. This is supported here experimentally by the increase in accuracy across all conditions when provided with the binary diagnosis, including % confidence of the system in its decision. The exception of this benefit is noted when the AI is incorrect and in pathological cases within the student group only, however this decrease in accuracy was small (-3.4%).

The reason for the increased in accuracy following binary diagnosis is not immediately clear, although may be related to the timing of the AI feedback, with the provision of the binary diagnosis, by necessity, at the end of the examination when the participant will have viewed all images. This may be the case in the clinical situation. A study by Gaube et al. (2021) [36] found that there was no difference in participants’ (radiologists and non-expert physicians) tendency to follow advice whether from a human or AI source, despite indicating preference for the human-derived decision support. This was found to encourage confirmation and anchoring biases and indicate this may be due to the discursive nature of true human to human interactions which exist organically in the clinical setting. The user should, therefore, be encouraged to seek the advice of a decision support tool rather than its automatic presentation, therefore potentially reducing cognitive and automation biases.

### Decision switching

As noted above, in general, exposure to the heatmap caused the diagnostic accuracy of the participants to fall and increase again when presented with the AI binary diagnosis and % confidence of the system. This indicated that the participants made a negative decision switch when presented with the heatmap. Automation bias has been defined by Goddard et al. (2014) and Bond et al. (2018) [23,24] as the ‘changing of mind’ to a less correct response because of computer intervention. This will not be a problem in a perfect system, where the AI is always correct. A change of decision will always be positive, however, even the best systems in use today are less than 100% accurate or may have some inherent biases which the user should be mindful of.

As expected, there is a greater propensity of the study participants to change their mind in a positive direction following AI feedback when the model is correct. This is not a finding which is fully supported in other studies, where the degree of accuracy of the AI feedback provided was not related to the propensity of the user to follow the advice given [36]. Goddard et al. (2014) [23] found that experienced users were less likely to change their mind from their initial decision. This may mean that they are less likely to gain advantage from the use of the system, however it was not possible to elicit this detail in this study due to the broader experience ranges classifying the experience groups in this study. However, the radiographers were less likely to change their mind following the presentation of either type of AI feedback across all conditions and the students were more likely to reconsider their initial decision (Table 5a, Figs 17 and 18). Interestingly, the radiographers in this study benefitted more than the students from the AI feedback, with the greatest net change in accuracy in the ‘AI correct’ and ‘pathological’ conditions (Table 5b). This may be useful in radiography where radiographer reporting results in high diagnostic accuracies [30,37], although there may be a greater propensity to underdiagnose pathology (‘false negatives’)[29].

### Automation bias

In most conditions there was a positive impact from the AI feedback despite the poor performance of the model. The heatmaps were more likely to cause the user to be unsure of their diagnosis but, overall, the net effect of the AI feedback on diagnostic accuracy was positive in both student and radiographer groups. The exception was in the case where the AI was incorrect, where the AI had a negative impact on the participants’ accuracy. A greater impact was seen in the student group where accuracy fell by 9.6% compared with 4.3% amongst radiographers, suggesting in this sample that the student group is more susceptible to automation bias (Table 5b). This has been found in other studies where the prevalence and likelihood of automation bias and decision switching is greater in less experienced clinicians [23,24].

#### Trust.

As reported in other studies, the qualified radiographers had a lower level of trust in AI than students. This may cause them to become anchored to their initial decision [24,36]. It could be assumed that those who were in the radiographer group were, on average, older than those in the student group. Generation Z (born mid 1990s – mid 2010s) are more likely to trust technology but also are more likely to be able to recognise the potentials and pitfalls of the technology that they are using [20]. This age group are also more likely to expect computer assistance in many avenues of their life with work being no exception [38]. Both groups’ trust perception in AI systems fell following participation in the study, perhaps indicating that they were able to detect that the AI, or some aspects of the AI were inaccurate, however, this is at odds with the increased accuracy reported above and with other studies indicating that even experienced clinicians are unable to detect inaccuracies in decision support systems [36].

For staff to develop appropriate trust in AI systems as used in clinical practice, it may be beneficial to have some degree of exposure to situations where the AI is incorrect. Example cases, where the AI is not always correct, such as those reported here, may be useful. Users should be exposed to cases which highlight the potential weaknesses in the system in order to calibrate trust. Cases presented by equipment manufacturers/software developers may not be those where the AI is not performing well and therefore appropriate trust cannot be calibrated by the user. The common benchmark of 30/70 split of correct/incorrect cases has been shown to allow users of technology to determine appropriate trust, and to neither over nor under-rely on the system [23,39]. This split should be considered when training new users of AI systems for clinical decision support.

## Limitations

There were a relatively small number of participants interpreting each examination, however this was intentional to encourage participation in an acceptable time frame, reducing the within-study attrition rate. There were 21 examinations included in this study. This number was chosen to provide exposure across a range of examinations, without having to specifically select examinations, therefore potentially introducing bias.

Convenience sampling was used to recruit the maximum number of participants. However, this sampling method can mean that the results are not generalisable to the wider profession. Additionally, this means of sampling resulted in more students than radiographers participating in this study. The reason for this is not clear, although it should be noted that this may skew the findings. The ‘student’ and ‘radiographer’ groups were analysed separately in an attempt to partially mitigate against this; however, future study should adopt purposive sampling of a wide experience range.

There was a lack of granular analysis of the levels of experience of the participants. This was to allow for a greater number of decision points for each interpretation. Furthermore, there is a lack of information gathered on the participants’ work experience/history and duration of such experience, which may impact the findings. This should be further investigated in future studies.

Examinations were not presented on high quality ‘reporting monitors’ as would have been the case in the clinical environment. Participants were able to access the study on any device of their choosing. This was due to constraints of conducting an experimental study during restrictions arising from the COVID pandemic. This may explain why the participants’ diagnostic accuracies are lower than reported in the literature. Further study should consider determining the impact of the difficulty of the examination on the impact of AI feedback from participants from different experience levels and clinical backgrounds.

## Conclusions

Radiographers’ and student radiographers’ accuracy in diagnosis can be improved with the use of AI, even a poorly functioning system. Participants in this study tended to follow the diagnosis from the system, resulting in decreased accuracy in the diagnostic task in some cases. This indicated that more education should be provided to undergraduate radiographers and other clinicians undertaking radiographic image interpretation.

Appropriate trust should be reached through exposure to imperfect AI. Trust in this imperfect AI decreased following exposure to feedback from the system, indicating that the user was aware of its fallibility. Biases inherent in both the model and the user will exist and maximum benefit can be derived from acknowledgement of both.

AI will be beneficial in, for example, diagnostic accuracy and workflow efficiencies when used appropriately in synchronicity with the clinician. This will be possible when the user can appreciate cases where the AI is incorrect or not useful. Knowledge of the strengths and weaknesses of the system will allow the clinician to determine its appropriateness for use in each task.

## Supporting information

S1 File Characteristics of the AI performance, study transcript, and findings from ANOVA tests with post-hoc pairwise comparisons. (DOCX)
