# Supplementary material for: The impact of AI feedback on the accuracy of diagnosis, decision switching and trust in radiography
Source: PLoS One. 2025 May 9;20(5):e0322051. doi: 10.1371/journal.pone.0322051 (PMC12064023; doi:10.1371/journal.pone.0322051)
Supplement: S1 File — (DOCX) [file pone.0322051.s001.docx]

**S1 - SUPPORTING INFORMATION**

**Characteristics of the AI performance**

| **AREA** | **PATIENT NUMBER (in QUALTRICS®)** | **CASE NO** | **AI PREDICTION / DIAGNOSIS** | **AI PROBABILITY** | **HUMAN EXPERT GROUND TRUTH** | **HUMAN:AI AGREEMENT** |
| --- | --- | --- | --- | --- | --- | --- |
| **elbow** | 1 | 141 | 0 | 99.83% | 0 | y |
| **elbow** | 2 | 184 | 0 | 99.32% | 1 | n |
| **elbow** | 3 | 329 | 0 | 99.54% | 0 | y |
| **finger** | 4 | 252 | 0 | 98.69% | 1 | n |
| **finger** | 5 | 290 | 0 | 97.35% | 1 | n |
| **finger** | 6 | 367 | 0 | 92.92% | 0 | y |
| **forearm** | 7 | 225 | 1 | 52.60% | 0 | n |
| **forearm** | 8 | 339 | 1 | 72.49% | 1 | n |
| **forearm** | 9 | 340 | 1 | 94.66% | 1 | y |
| **hand** | 10 | 1 | 1 | 56.85% | 0 | n |
| **hand** | 11 | 101 | 1 | 83.60% | 1 | y |
| **hand** | 12 | 103 | 1 | 65.24% | 0 | n |
| **humerus** | 13 | 120 | 0 | 53.51% | 0 | y |
| **humerus** | 14 | 199 | 1 | 55.80% | 1 | y |
| **humerus** | 15 | 321 | 0 | 59.11% | 1 | n |
| **thumb** | 16 | 66 | 0 | 96.98% | 0 | y |
| **thumb** | 17 | 128 | 0 | 91.79% | 0 | y |
| **thumb** | 18 | 370 | 0 | 97.79% | 0 | y |
| **wrist** | 19 | 98 | 0 | 79.94% | 0 | y |
| **wrist** | 20 | 124 | 0 | 56.13% | 1 | n |
| **wrist** | 21 | 221 | 0 | 88.81% | 0 | y |
| ***AI accuracy (agreement with ground truth (established from three to five reporting radiographers and radiologists): 12 cases where the AI agreed with ground truth /21 total cases: AI accuracy: 57.1%)*** | | | | | | |

**Study transcript**

**ANOVA with post-hoc pairwise comparisons**

| ***Condition*** | ***Within subject effect (repeated measure), Combined (measure+exp)*** | ***n= (stud, rad)*** | ***Mean (stud, rad)*** | ***Std. dev.*** | ***Box's test of equality of co-variance matrices*** | ***Levene's test (univariate)*** | ***Mauchly's test of sphericity*** | ***F-ratio*** | ***Degrees of freedom (dfM, dfR)*** | ***Significance p=…*** | ***Effect size (partial eta squared)*** | ***Pairwise comparisons*** |
| --- | --- | --- | --- | --- | --- | --- | --- | --- | --- | --- | --- | --- |
| ***ALL*** | Within subjects (repeated measures) i.e. preHM, postHM, post bin | 21, 21 | PreHM 49.9121, 57.4027.  PostHM 45.5789, 57.5161.  PostBin 54.2593, 64.8753 | PreHM 23.07891, 23.06881. PostHM 22.92437, 25.84600. PostBin 31.29149, 34.33021 | p=0.863 Equality of variance assumed | PreHM p=0.834, PostHM p=0.828, PostBin p=0.828 | <0.001 Sig (sphericity violated) | (G-G correction) F=2.649 | 1.452, 58.085 | **0.095**  *Significant at α = .10* | **0.062 (medium effect size)** | *Visual inspection – no pattern* |
|  | Combined effect |  |  |  |  |  |  | G-G correction F= 0.649 | 1.452, 58.085 | 0.748 |  |  |
| ***AI correct*** | Within subjects (repeated measures) | 13, 13 | PreHM 44.3341, 50.5196. PostHM 40.8124, 52.6175. PostBin 57.2558, 65.2381 | PreHM 22.79231, 16.20699. PostHM 21.51564, 25.23205. PostBin 28.24029, 28.31949 | p=0.540 Equality of variance a  ssumed | Pre HM p=0.133, Post HM p=0.515, Post Bin p=0.600 | p=0.017 sig | Huyhn-Feldt used (GG=0.771) F=7.823 | 1.696, 40.709 | **0.002** | **0.246 (large effect)** | **PreHM and PostBin p=0.007 ( CI -24.220 - -3.410, mean difference -13.815)** |
|  | Combined effect |  |  |  |  |  |  | F=0.239 | 1.696, 40.709 | 0.752 |  |  |
| ***AI incorrect*** | Within subjects (repeated measures) | 8, 8 | PreHM 58.9762, 68.5714. PostHM 53.3244, 65.4762. PostBin 49.3899, 64.2857 | PreHM 21.89948, 28.89119. PostHM 24.43698, 26.46180. PostBin 37.23777, 44.63000 | p=0.417 Equality of variance assumed | PreHM p=0.816, PostHM p=0.611, PostBin p=0.275 | p=0.004 sig | GG correction F=0.631 | 1.268, 17.750 | 0.474 |  |  |
|  | Combined effect |  |  |  |  |  |  | F=0.090 | 1.268, 17.750 | 0.825 |  |  |
| ***Pathological*** | Within subjects (repeated measures) | 9, 9 | PreHM 63.2860, 68.4061. PostHM 50.6911, 64.5437. PostBin 59.8942, 84.8148 | PreHM 22.77461, 28.05400. PostHM 30.65670, 30.06630. PostBin 37.96613, 22.79809 | p=0.417 Equality of variance assumed | PreHM p=9.553, PostHM p=0.849, PostBin p=0.072 | p=0.012 sig | GG correction F=6.252 | 1.381, 22.097 | **.013** | **0.281 (large effect)** | **PreHM and PostHM p=0.015 (CI 1.457 – 15.001, mean diff 8.229) AND PostHM and PostBin p=0.013 (CI 2.984 – 26.580, mean diff 14.737)** |
|  | Combined effect |  |  |  |  |  |  | F=2.882 | 1.381, 22.097 | **.097**  *Significant at α = .10* | **0.150 (large effect)** | *Visual inspection – no pattern* |
| ***Non-pathological*** | Within subjects (repeated measures) | 12, 12 | PreHM 39.8816, 49.1501. PostHM 41.7447, 52.2454. PostBin 50.0331, 49.9206 | PreHM 18.30592, 14.89435. PostHM 15.31557, 22.05492. PostBin 26.20137, 34.57704 | p=0.417 Equality of variance assumed | PreHM p=0.480, PostHM p=0.382, PostBin p=0.125 | p=0.003 sig | GG correction F=0.532 | 1.405, 30.906 | .531 not sig |  |  |
|  | Combined effect |  |  |  |  |  |  | F=0.599 | 1.405, 30.906 | .499 |  |  |
